# Editorial: Rising stars in pediatric pulmonology

**DOI:** 10.3389/fped.2025.1627194

**Published:** 2025-06-11

**Authors:** Kate Ching-ching Chan, Gabrielle B. McCallum, Javier Rodriguez-Fanjul, Elpis Hatziagorou

**Affiliations:** ^1^ Department of Paediatrics, The Chinese University of Hong Kong, Shatin, Hong Kong SAR, China; ^2^ Laboratory for Paediatric Respiratory Research, Li Ka Shing Institute of Health Sciences, Faculty of Medicine, The Chinese University of Hong Kong, Shatin, Hong Kong SAR, China; ^3^ Hong Kong Hub of Paediatric Excellence, The Chinese University of Hong Kong, Kowloon, Hong Kong SAR, China; ^4^ Child and Maternal Health Division, Menzies School of Health Research, Charles Darwin University, Darwin, NT, Australia; ^5^ School of Nursing and Midwifery, Faculty of Health, Charles Darwin University, Darwin, NT, Australia; ^6^ Pediatric Intensive Care Unit, Hospital Germans Trias I Pujol, Universitat Autonoma de Barcelona, Badalona, Spain; ^7^ Pediatric Pulmonology Unit, 3rd Pediatric Department, Hippokration Hospital of Thessaloniki, Aristotle University of Thessaloniki, Thessaloniki, Greece

**Keywords:** pediatric pulmology, asthma, respiratory distress syndrome, bronchopulmonary dysplasia, lung injury

**Editorial on the Research Topic**
Rising stars in pediatric pulmonology

Pediatric pulmonology is a specialized field dedicated to understanding and managing respiratory problems in children. Ongoing research in pediatric pulmonology is crucial in advancing our knowledge of these conditions, refining treatment protocols, and ultimately improving the quality of life for affected children. To ensure sustained innovation and progress in this field, it is imperative to foster the development of emerging researchers. This special edition presents a Research Topic of four new research articles from international researchers. Each article broadens our understanding of pediatric pulmonology, from bench to bedside, covering various conditions including respiratory distress syndrome (RDS), bronchopulmonary dysplasia (BPD), lung injury and asthma.

Advancements in neonatal intensive care have significantly improved the survival rates of preterm infants. Despite these advancements, RDS remains one of the leading causes of morbidity and mortality in this population (1). Surfactant therapy and non-invasive ventilation are proven effective treatments for enhancing outcomes in this group. Less-invasive surfactant administration (LISA) has been accepted as the preferred method of surfactant delivery for spontaneously breathing preterm infants receiving continuous positive airway pressure (CPAP) (2). LISA has shown better outcomes than surfactant administration via an endotracheal tube (ETT) (3). However, CPAP failure after LISA, defined as the need for intubation within the first 72 h after birth, is not uncommon. Therefore, meticulous consideration of individual patient factors is paramount when developing the optimal management strategy. Alsina-Casanova et al.'s study investigated predictors of CPAP failure in preterm infants with RDS after LISA. The study included preterm infants born between 23 and 33 weeks gestational age in two level III Neonatal Units. CPAP failure occurred in 21.8% of LISA patients. Lower gestational age, intrauterine growth restriction, lower admission temperature, lower saturation/fraction of inspired oxygen (SF) ratio, and higher lung ultrasound (LUS) score were the best predictors of CPAP failure after LISA. A predictive model was constructed using these factors. The model demonstrated an area under the curve of 0.84. Clinicians should consider risk factors for CPAP failure when selecting patients for LISA. LUS and SF ratio at admission may aid this decision-making process.

Bronchopulmonary dysplasia (BPD) is a neonatal lung disease that causes substantial morbidity and mortality in preterm newborns. Its pathogenesis is complex, and therapeutic options are limited. The review article by Yang et al. elucidates the role of protein post-translational modifications (PTMs) in the development of BPD. PTMs, such as phosphorylation, acetylation, ubiquitination, SUMOylation, methylation, glycosylation, glycation, S-glutathionylation, and S-nitrosylation, play potential roles in the molecular mechanisms underlying the pathogenesis of BPD. PTMs regulate cellular functions by altering the characteristics of substrate proteins in response to environmental changes. Understanding the interactions and influences of different PTMs on BPD is crucial for establishing comprehensive diagnostic markers and therapeutic targets. The review underscores the significance of PTMs in the pathogenesis of BPD and suggests that further research is necessary to comprehend the crosstalk between different PTMs and their effects on various substrate proteins. This knowledge could revolutionise the approach to managing BPD by providing novel insights into its molecular mechanisms and identifying novel therapeutic targets.

Traffic accidents, especially blunt impacts, cause severe injuries in children (4). Wang et al. assessed inflammatory and injury responses in infant rabbits with acute lung injury from blunt impact. Lung tissue exhibited alveolar wall destruction, along with edema and granulocyte infiltration in the pulmonary interstitial tissue and alveolar cavities. Blood white blood cell count and neutrophil percentage increased significantly and decreased after 24 and 72 h. The lung wet/dry weight ratio showed significant oedema, corroborated by histopathology. Surfactant protein A (SP-A) levels decreased immediately post-injury but gradually recovered between 24 and 72 h. Interleukin-6 (IL-6) and Interleukin-8 (IL-8) increased rapidly post-injury, which may contribute to the progression of acute lung injury to acute respiratory distress syndrome. SP-A, synthesised and secreted by alveolar epithelial cells, may serve as a biomarker indicating alveolar injury and repair, providing insights into the severity of lung injury.

Asthma is a prevalent chronic disease with substantial global health impacts. It is a complex inflammatory disease of the airways, characterised by various pathophysiological features. Although the exact aetiology of asthma remains largely undefined, it is believed that genetic predisposition, environmental influences, and their interactions play a pivotal role in the pathogenesis of asthma (5). Iron deficiency anaemia (IDA) has been proposed to influence asthma, although the causal relationship remains uncertain (6). This study by Li et al. employed Mendelian randomization (MR) to investigate the causal link between IDA and asthma. Five single nucleotide polymorphisms (SNPs) were utilised as genetic markers for exposure factors. Genetically determined IDA was found to be significantly associated with an elevated risk of asthma. This study suggests that IDA may be associated with a higher risk of asthma, thereby underscoring the necessity for further research into the underlying mechanisms involved. Additionally, it is pertinent to assess whether addressing IDA could potentially reduce asthma incidence and enhance management in paediatric populations.

In conclusion, this Research Topic provides valuable new insights into significant pediatric respiratory conditions, including RDS, BPD, lung injury, and asthma. We acknowledge the contributions of emerging researchers whose work is featured here, as their innovative research paves the way for future advancements in pediatric pulmonology.
